# Genetic variants in hypoxia‐inducible factor pathway are associated with colorectal cancer risk and immune infiltration

**DOI:** 10.1111/jcmm.18019

**Published:** 2023-11-23

**Authors:** Mengfan Guo, Jie Lin, Xiangming Cao, Jieyu Zhou, Shuai Ben, Silu Chen, Haiyan Chu, Lin Miao, Shuwei Li, Dongying Gu

**Affiliations:** ^1^ Department of Oncology, Nanjing First Hospital Nanjing Medical University Nanjing China; ^2^ Department of Environmental Genomics, Jiangsu Key Laboratory of Cancer Biomarkers, Prevention and Treatment, Collaborative Innovation Center for Cancer Personalized Medicine Nanjing Medical University Nanjing China; ^3^ The Affiliated Cancer Hospital of Nanjing Medical University Jiangsu Cancer Hospital, Cancer Institute of Jiangsu Province Nanjing China; ^4^ Department of Oncology The Affiliated Jiangyin Hospital of Nantong University Wuxi China; ^5^ Department of Genetic Toxicology, The Key Laboratory of Modern Toxicology of Ministry of Education, Center for Global Health, School of Public Health Nanjing Medical University Nanjing China; ^6^ Medical Center for Digestive Diseases The second Affiliated Hospital of Nanjing Medical University Nanjing China

**Keywords:** colorectal cancer, genetic variants, HIF, hypoxia, immune status

## Abstract

Hypoxia‐inducible factor (HIF) pathway genes influence tumorigenesis and immune status. However, the associations between genetic variants in hypoxia‐related genes and colorectal cancer risk and the immune status of hypoxia‐associated genes in colorectal cancer have not been systematically characterized. The associations between genetic variants and colorectal cancer risk were evaluated in Chinese, Japanese and European populations using logistic regression analysis. The relationships between target genes and tumour immune infiltration were predicted by Tumour Immune Estimation Resource (TIMER). We found that rs34533650 in *EPAS1* was associated with colorectal cancer risk (OR = 1.43, 95% CI = 1.20–1.70, *P*_(FDR)_ = 8.35 × 10^−4^), and this finding was validated in two independent populations (Japanese: OR = 1.07, 95% CI = 1.01–1.15, *p* = 3.38 × 10^−2^; European: OR = 1.11, 95% CI = 1.03–1.19, *p* = 6.04 × 10^−3^). *EPAS1‐*associated genes were enriched in immune‐related pathways. In addition, we found that EPAS1 copy number variation (CNV) was associated with the degree of infiltration of immune cells and observed correlations between *EPAS1* expression and immune cell infiltration levels in colorectal cancer. These results highlight that genetic variants of hypoxia‐related genes play roles in colorectal cancer risk and provide new insight that *EPAS1* might be a promising predictor of colorectal cancer susceptibility and immune status.

## 1 INTRODUCTION

Colorectal cancer presents a major health burden worldwide,^1^ attracts attention due to its high incidence and mortality rates,^2^ and presents an early‐onset trend.^3^ The number of colorectal cancer patients in China is rapidly increasing,^4^ mainly due to the interaction of genes and the environment.^5^ Through genome‐wide association studies (GWASs), multiple independent variants in colorectal cancer have been identified,^6^ and most of the variants are believed to act by regulating gene expression.^7^ Genetics and immunity play an important role in colorectal cancer.^8^

Hypoxia activates several adaptive responses, which are mainly mediated by HIF1α and HIF2α.^9^ Previous studies suggested that hypoxia‐induced changes affect tumour immune microenvironment.^10^ Hypoxia‐inducible factor (HIF) pathway genes influence the expression of gene in both tumour cells and immune cells and affect tumour progression and treatment response.^11^ The HIF pathway, including *HIF‐1α*, *EPAS1*, *HIF‐3α* and *ARNT*, is associated with disease progression and adverse clinical outcomes in colorectal cancer.^12^ EGLN1, EGLN2 and EGLN3 regulate the activity of HIF‐1α and EPAS1, which are widely expressed in cells.^13^ The loss of Von Hippel–Lindau (VHL) impacts the stabilisation of HIF‐1α and EPAS1 and activates the signals that mediate adaptive responses.^14^ *EPAS1* encodes a subunit of HIFs^15^ and shows multifaceted effects, including regulation of angiogenesis, haemoglobin concentration and erythrocytosis.^16^ *EPAS1* affect immune infiltration in paraganglioma and pheochromocytoma.^9^ The immune microenvironment can be used to predict the treatment outcomes and curative effects,^17^ and the treatment response and prognosis of colorectal cancer are associated with the immune microenvironment.^18^ In addition, the outcome and progression of many types of diseases are affected by single nucleotide polymorphisms (SNPs),^19^ and studies have demonstrated that SNPs in *HIF‐1α* are associated with susceptibility to multiple cancers.^20^ However, few studies have systematically elucidated the correlations between genetic variants in HIF pathway genes and the susceptibility of colorectal cancer.

In this study, we aimed to assess the relationships between SNPs in HIF‐associated genes and colorectal cancer risk in a discovery set and two validation sets and elucidate possible immunoregulatory mechanisms.

## 2 MATERIALS AND METHODS

### 2.1 Study subjects

In the discovery study, 1150 colorectal cancer cases and 1342 controls were recruited in the First Affiliated Hospital of Nanjing Medical University and the Affiliated Nanjing First Hospital. Subjects were excluded due to unexpected duplications or genetic relatedness. All of subjects were frequency‐matched based on age and sex and signed written informed consent. Cases were diagnosed with colorectal cancer via pathological examination, and patients with previous cancer were excluded. Unqualified subjects were excluded based on physical examination. Questionnaires to collect information on demographic factors were administered through face‐to‐face interviews. This research was approved by Nanjing Medical University Institutional Review Board.

In addition, summary estimates from the Japanese population [(6692 cases from the BioBank Japan Project (BBJ) and 27178 controls from JPHC (Japan Public Health Center), J‐MICC (Japan Multi‐Institutional Collaborative Cohort) and ToMMo (Tohoku Medical Megabank Organization)] and genotyping data from the European population [4461 cases and 4140 controls from Genetics and Epidemiology of Colorectal Cancer Consortium (GECCO) deposited in the database of Genotypes and Phenotypes (dbGaP) phs001315.v1.p1] were used to verify the results of the Chinese population. Individuals without expected relatedness were retained for analysis.^21^, ^22^ Written informed consent was given by all recruited patients, and the projects were approved by the relevant research ethics committees at each institute. The characteristics of the three groups are shown in Table S1.

### 2.2 Selection of genes

After screening reported studies, we selected critical genes involved in the HIF pathway. The HIF pathway consists of a family of transcription factors HIFs and their regulatory proteins.^23^ HIFs contain two different subunits: α and β. The α part composes of HIF‐1α/HIF1A, HIF‐2α/EPAS1 and HIF‐3α/HIF3A; the β part contains two protein HIF‐1β/ARNT1 and HIF‐2β/ARNT2.^24^ The regulatory proteins include the prolyl hydroxylase domain enzymes (PHDs), VHL and factor inhibiting HIF (FIH, also known as HIF1α subunit inhibitor, HIF1AN).^23^, ^25^ The PHDs include PHD1/EGLN1, PHD2/EGLN2 and PHD3/EGLN3,^26^ and the VHL protein binds to CUL2, ELOB, ELOC and Rbx1 to regulate HIF.^27^ HIF1AN prevents the recruitment of the coactivators EP300 and CREB‐binding protein (CREBBP).^28^ Finally, a total of 16 genes were used selected in the HIF pathway.

The differentially expressed genes were identified using The Cancer Genome Atlas (TCGA) database and met the following criteria: (a) call rate > 95%; (b) fold change >1.5 and (c) *p* < 0.05. To further identify the genes associated with colorectal cancer, we performed principal component analysis (PCA) and orthogonal partial least square‐discriminate analysis (OPLS‐DA) on HIF‐related genes using data from the TCGA database.

### 2.3 Selection and genotyping for SNPs

According to the 1000 Genomes Project, SNPs located in selected genes were mapped to the Chinese Han population in Beijing (CHB) data. Selected SNPs met the following criteria: (a) call rate > 95%; (b) minor allele frequency (MAF) ≥ 0.05 and (c) Hardy–Weinberg equilibrium (HWE) ≥ 0.05. Through pairwise linkage disequilibrium (LD) analysis, tagged SNPs were extracted (*r* ^2^ ≤ 0.6). The functions of SNPs were predicted by RegulomeDB (http://regulomedb.org/) and HaploReg v4.2 (https://pubs.broadinstitute.org/mammals/haploreg/haploreg.php). The SNPs were reserved with Regulome DB Score < 6, histone marks or motif changes, implying that variants had potential functions.

Genomic DNA was isolated from peripheral blood samples. For genotyping, Illumina Human Omni ZhongHua Bead Chips were used. Eighteen SNPs were included after removing the LD redundancy and functional annotation in the genotyping stage.

### 2.4 Functional analysis

The gene‐based analysis was performed by Versatile Gene‐based Association Study 2 (VEGAS2, https://vegas2.qimrberghofer.edu.au/). The CancerSplicingQTL database (http://www.cancersplicingqtl‐hust.com/#/) was used to predict the splicing of SNPs. Enhancer states of SNPs were predicted through 3DSNP v2.0 (https://omic.tech/3dsnpv2/). To explore associations between variants and various traits, we used the MRC‐IEU OpenGWAS database (https://gwas.mrcieu.ac.uk/).^29^

Oncomine (https://www.oncomine.org) was used to assess gene expression in multiple tumour tissues. The differences in mRNA expression levels were calculated using the TCGA dataset, Gene Expression Omnibus (GEO) database and in‐house RNA‐Seq data.

### 2.5 Prediction of target genes at single‐cell level

The single‐cell data was used for analysis to explain the functional states of cancer cells. At the single‐cell level, gene expression was predicted using colorectal tissues in The Human Protein Atlas (HPA, https://www.proteinatlas.org/). The role of target genes in 14 crucial functional states was predicted by the CancerSEA database (http://biocc.hrbmu.edu.cn/CancerSEA/).

### 2.6 TME immunoregulation analysis

Immunoregulation analysis was performed using the Tumour Immune Estimation Resource (TIMER, https://cistrome.shinyapps.io/timer/).^30^ The correlations between target gene expression and the abundance of TME‐infiltrating immune cell types were evaluated. The correlations between immune markers in immune cell types and target gene were also assessed. Adjustments were made for tumour purity, which has been proven to influence the analysis of immune infiltration.

### 2.7 Statistical analysis

To assess the differences between colorectal tumour tissues and normal tissues, the PCA model was used. The variable importance in the projection (VIP) value was calculated to indicate the contribution of genes to the classification using the OPLS‐DA model, and the VIP value >1.0 was considered significant. The quality of the OPLS‐DA model was based on the parameter R^2^, which is known as the goodness of fit of the model (cut‐off value ≥0.50). Unconditional logistic regression models were used to assess the associations between genetic variants and colorectal cancer risk, with adjustment for age, sex, PC1 and PC2 (where appropriate). For correction, the false discovery rate (FDR) method was used. The associations between different haplotypes and colorectal cancer risk were assessed by haplotype analysis. The heterogeneity of different studies was evaluated using *I* ^2^, and *I* ^2^ provides an estimate of the amount of variance. Multiplicative and additive effects were used to assess the interaction effects between gene–environment interactions. The effects of genetic variants on the expression of genes were performed by the expression quantitative trait loci (eQTL) analysis. The differences in candidate gene mRNAs (log2 transformed) were compared using the TCGA dataset, GEO database and in‐house data, and the gene expression differences were estimated using Student's *t*‐test. Cox regression analyses were used to evaluate the associations between *EPAS1* expression and the overall survival (OS) of patients with colorectal cancer. To investigate the potential mechanisms of target genes, gene set enrichment analysis (GSEA) was used. All analyses were performed with R 4.0.2, PLINK 1.90 and SIMCA software (version 13.0). *p* < 0.05 was considered statistically significant.

## 3 RESULTS

### 3.1 Associations of candidate SNPs with colorectal cancer risk

**FIGURE 1:**
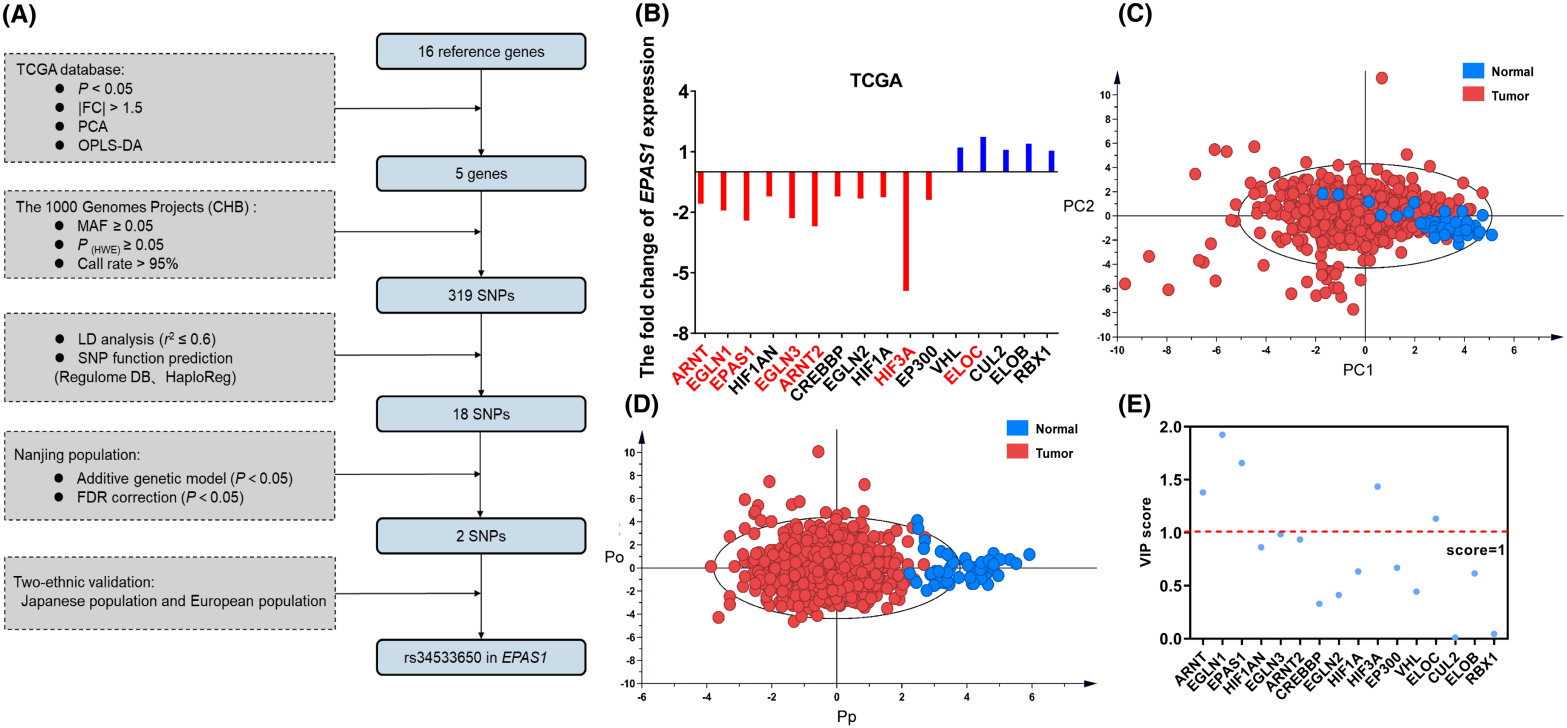
The procedures of selecting colorectal cancer functional genetic variants in the HIF pathway. (A) The flow chart for selecting potential functional genetic variants. (B) The fold change in the expression of key HIF pathway genes. (C) Principal component analysis (PCA). (D) Orthogonal partial least squarediscriminant analysis (OPLS‐DA). Pp represents the predictive component, and Po represents the orthogonal component. (E) Scatter plot from the OPLS‐DA model using the variable influence on projection (VIP) score. All data were obtained from the TCGA database. FC, fold change; CHB, Han Chinese in Beijing; MAF, minor allele frequency; HWE, Hardy–Weinberg equilibrium; LD, linkage disequilibrium; SNP, single nucleotide polymorphism; FDR, false discovery rate.

Figure 1A shows the process of this study. Seven differentially expressed genes were identified among these 16 key genes using TCGA database, as shown in Figure 1B. PCA and OPLS‐DA were used to identify the genes associated with colorectal cancer. Notably, the adjacent mucosa was separated from tumour samples (Figure 1C). Clustering between these two groups was shown through an OPLS‐DA score plot (Figure 1D), reflecting many differentially expressed genes between them. Finally, five genes (VIP value >1.0) contributed to the difference between the two groups and were differentially expressed between them (Figure 1E).

After a systematic process of screening SNPs, a total of 18 SNPs in five cancer‐related genes were obtained to explore the associations with colorectal cancer risk. The functions of these SNPs are shown in Tables S2. There were five SNPs associated with susceptibility to colorectal cancer in an additive model. After FDR correction, only two SNPs increased the susceptibility to colorectal cancer (rs34533650: *P*_(FDR)_ = 8.35 × 10^−4^, rs6753127: *P*_(FDR)_ = 5.91 × 10^−3^, Table S3).

Notably, rs34533650 and rs6753127 were located in the *EPAS1* gene in low disequilibrium (*r* ^2^ ≤ 0.6). These two SNPs were combined to calculate the number of risk alleles. As shown in Table S4, individuals carrying 1 and 2–4 risk alleles had increased colorectal cancer risk [odds ratio (OR) = 1.86, confidence intervals (95%CI) = 1.55–2.22; OR = 1.78, 95% CI = 1.25–2.54, respectively]. Additionally, with the increasing number of risk alleles, the susceptibility to colorectal cancer accumulated (*P*_trend_ = 6.59 × 10^−11^). Compared with the most common haplotype AC, the haplotype GC in the *EPAS1* gene increased colorectal cancer risk (OR = 1.48; 95% CI = 1.23–1.78, in Table S5).

We subsequently evaluated the two SNPs in two independent validations. As shown in Table 1, rs34533650 increased colorectal cancer risk in the Japanese and European populations (OR = 1.07, 95% CI = 1.01–1.15, *p* = 3.38 × 10^−2^; OR = 1.11, 95% CI = 1.03–1.19, *p* = 6.04 × 10^−3^, respectively), and the meta‐analysis showed the same trend (OR = 1.16, 95% CI = 1.03–1.30, *p* = 1.17 × 10^−2^). The MAF values of the East Asian and the European population in the 1000 Genomes Project were consistent with the MAF of the Chinese population and European population in this study Table S6). To reveal the associations between the expression levels of *EPAS1* and these two SNPs, the eQTL analysis was conducted. However, the mutant genotype of rs34533650 tended to decrease *EPAS1* expression using in‐house database (*p =* 2.01 × 10^−6^, Figure S1). Similarly, the most significant correlation existed between SNP rs34533650 and susceptibility to colorectal cancer through the gene‐based analysis in Table S7.

**TABLE 1:** Association analyses between rs34533650 in *EPAS1* and colorectal cancer risk in discovery and validation stages.

Stages	Population	Cases/controls	rs34533650 genotypes (AA/AG/GG)		MAF	*P*	*P* ^a^	OR (95% CI)^a^	*P* ^b^
			Cases	Controls	Case/control				
Discovery	Chinese	1,150/1,342	841/288/21	1,022/227/19	0.14/0.10	4.64 × 10^−5^	1.43 (1.20–1.70)	4.92 × 10^−5^	
Validation‐1	Japanese	6,692/27,178	NA	NA	NA	3.38 × 10^−2^	1.07 (1.01–1.15)	3.38 × 10^−2^	
Validation‐2	European	4,461/4,140	2,587/1,603/271	2,513/1,418/209	0.24/0.22	6.04 × 10^−3^	1.11 (1.03–1.19)	6.04 × 10^−3^	
	Combined^c^	12,303/32,660				1.17 × 10^−2^	1.16 (1.03–1.30)	1.17 × 10^−2^	8.60 × 10^−3^

*Note*: Some controls are missing in the discovery stages.

Abbreviations: CI, confidence interval; NA, not available; OR, odds ratio.

^a^ *p*‐value of the additive model with adjustment for age, sex, PC1 and PC2 (where appropriate) in the logistic regression model.

^b^ *p‐*value for heterogeneity.

^c^ A meta‐analysis was performed for combined group.

### 3.2 Association between rs34533650 and colorectal cancer risk

To analyse the associations between rs34533650 and colorectal cancer risk, four genetic models were used (Table 2). Compared with the AA genotype, rs34533650 AG/GG was markedly associated with the risk of colorectal cancer (OR = 1.53, 95% CI = 1.26–1.85, *p* = 1.40 × 10^−5^). There was no association between individuals carrying mutant genotypes and colorectal cancer risk (OR = 1.21, 95% CI = 0.64–2.26, *p* = 5.58 × 10^−1^, in the recessive model).

**TABLE 2:** Association analyses between rs34533650 in *EPAS1* and colorectal cancer risk in Chinese populations.

Genotypes	Cases	Controls	*P*	OR (95% CI)	*P* ^a^	OR (95% CI)^a^
	n (%)	n (%)				
AA	841 (73.13)	1,022 (80.60)		1.00		1.00
AG	288 (25.04)	227 (17.90)	3.57 × 10^−1^	1.34 (0.72–2.52)	3.56 × 10^−1^	1.34 (0.72–2.52)
GG	21 (1.83)	19 (1.50)	1.56 × 10^−5^	1.54 (1.27–1.88)	1.55 × 10^−5^	1.54 (1.27–1.88)
Additive model			4.64 × 10^−5^	1.43 (1.20–1.70)	4.92 × 10^−5^	1.43 (1.20–1.70)
Dominant model			1.38 × 10^−5^	1.53 (1.26–1.85)	1.40 × 10^−5^	1.53 (1.26–1.85)
Recessive model			5.29 × 10^−1^	1.22 (0.65–2.29)	5.58 × 10^−1^	1.21 (0.64–2.26)

Abbreviations: CI, confidence interval; OR, odds ratio.

^a^ *p*‐value of the additive model with adjustment for age, sex, PC1 and PC2 (where appropriate) in the logistic regression model.

In different subgroups, stratified analysis was performed to evaluate associations between genetic variants and the risk of colorectal cancer (Table S8 and S9). The mutant genotype associated with colorectal cancer risk in the subgroups of age, sex, smoking status, alcohol consumption status, tumour site, tumour grade and Dukes stage. We also performed interaction analysis on *EPAS1* rs34533650 and basic characteristics, and no significant multiplicative or additive interactions were observed. There were no statistically significant differences for the distributions of allele frequencies were observed in the tumour site, tumour grade and Dukes stage (Table S9).

### 3.3 Potential regulatory function of rs34533650

The annotation of rs34533650 is shown in Figure S2A using data derived from HCT116 cells, and the potential functions of the target SNP were predicted by in silico analysis. The SNP rs34533650 was located in the intron, which was considered to have enhancer states in 14 cell types (Table S10). There was no evidence to suggest that rs34533650 affects *EPAS1* splicing through the CancerSplicingQTL database (Table S2). *EPAS1* rs34533650 was bound to BARX1, POU4F2 and POU4F3 according to RegulomeDB (Figure S2B). After phenome‐wide association study (PheWAS) analysis for rs34533650, we found that the target SNP was associated with multiple diseases, as shown in Table S11.

### 3.4 Expression levels of *EPAS1*

**FIGURE 2:**
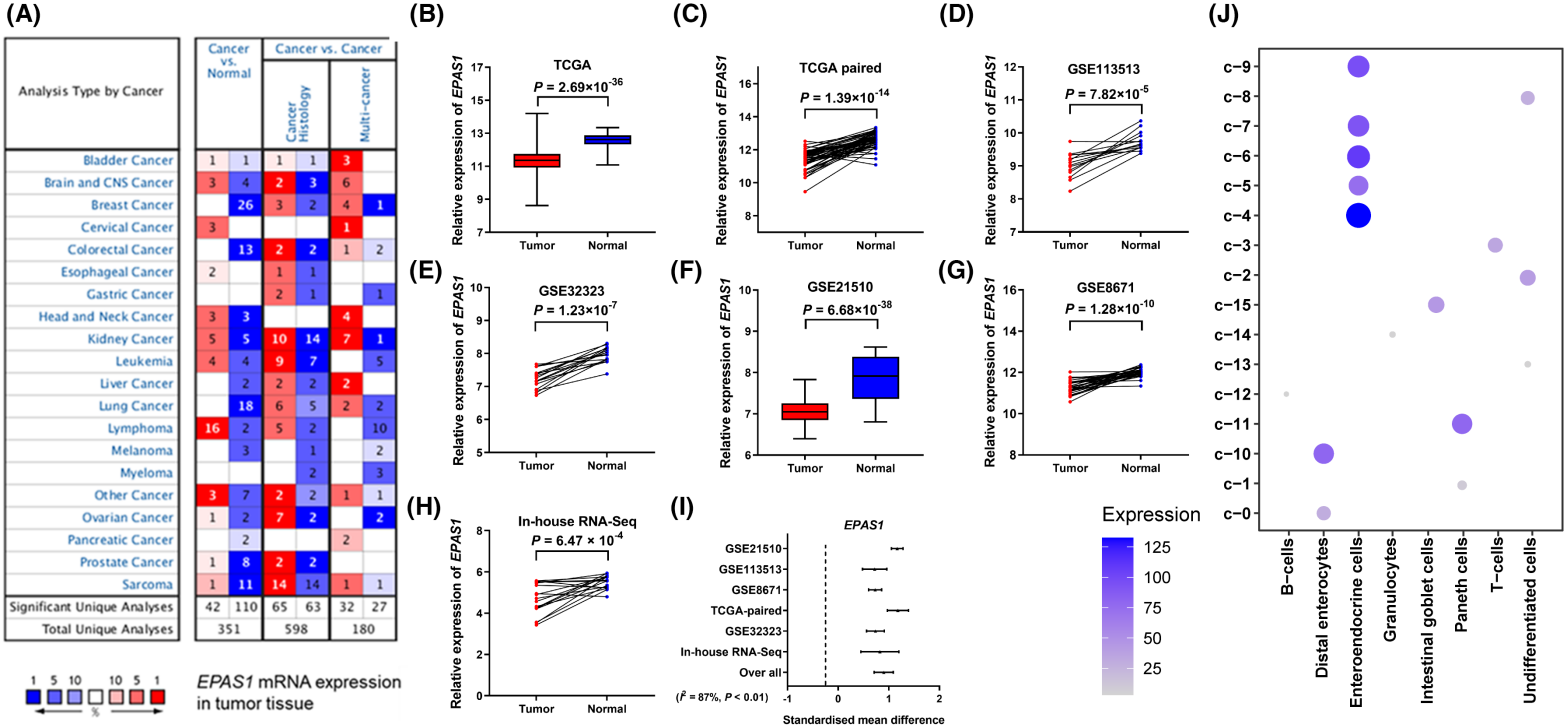
*EPAS1* had significantly lower  expression levels in colorectal tumour tissues than in normal tissues. *EPAS* *1* was highly expressed in multiple normal tissues through the Oncomine database, and numbers represent the number of datasets with gene expression differences in cancer versus normal tissues, upregulated (left column, red) and downregulated (right column, blue) (A), The *EPAS1* expression in the TCGA database (B‐C), GEO database (GSE113513, GSE32323, GSE21510 and GSE8671) (D‐G), and in‐house RNA‐Seq database (H). A meta‐analysis of the associations between *EPAS1* expression and colorectal cancer risk was performed on these six studies. The horizontal axis represents SMD and 95% CI (I). *EPAS1* expression in different cell types using single‐cell sequencing data from colorectal tissues in The Human Protein Atlas database (J).

According to the Oncomine database, *EPAS1* was low expressed in multiple tumour tissues in Figure 2A. The level of *EPAS1* expression was low in tumour tissues using the TCGA database, TCGA paired database, GSE113513, GSE32323, GSE21510, GSE8671 and in in‐house database (Figure 2B–H). Overall, the mRNA levels of *EPAS1* were significantly decreased in tumour tissues [standard mean deviation (SMD) = 0.90, 95% CI = 0.71–1.09), Figure 2I]. We predicted the role of *EPAS1* in crucial functional states in colon cancer at the single‐cell level and observed positive correlations between *EPAS1* and angiogenesis, quiescence, differentiation, epithelial‐mesenchymal transition (EMT), metastasis and hypoxia, as shown in Figure S3.

We also assessed the relationships between *EPAS1* expression and clinicopathological or demographic characteristics. *EPAS1* expression was lower in tumour samples (in GSE44076, Figure S4A). No significant difference was observed between subgroups of age (in GSE44076, Figure S4B). Compared with grade I or II, *EPAS1* was expressed at low levels in grade III (in the GSE17536 database, Figure S4C). *EPAS1* expression was significantly decreased in tissues with a high degree of lymph node involvement (in GSE21815, Figure S4D) and varied between different levels of malignant tissue in the GSE21510 database (Figure S4E), and we found similar phenomenon in the TCGA database (Figure S4F). These results indicated that *EPAS1* may be involved in tumour progression. The patients with low *EPAS1* expression had slightly shorter OS times than those with high levels of *EPAS1* expression, although the *p* value was larger than 0.05 (in GSE12945, Figure S5). *EPAS1* expression was enriched in enteroendocrine cells according to single‐cell sequencing data from colorectal tissues in the HPA database (Figure 2J).

### 3.5 Correlation between *EPAS1* expression and infiltration of immune cells

**FIGURE 3:**
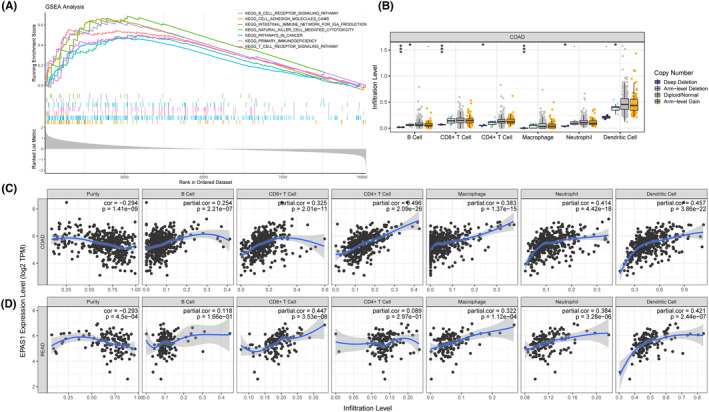
Immune infiltration analysis based on *EPAS1*. (A) GSEA based on *EPAS1* expression using the TCGA database. (B) EPAS1 CNV affects the infiltrating levels of B cells, CD8+ T cells, CD4+ T cells, macrophages, neutrophils and dendritic cells in COAD. (C‐D) Analysis of the associations between *EPAS1* expression and the levels of infiltrating immune cells in COAD and READ tissues based on TIMER.

Hypoxia was associated with the activity of immune cells in the tumour microenvironment. Based on the expression of *EPAS1*, multiple immune‐related gene sets and cancer‐related pathway were enriched in colorectal cancer through the GSEA analysis including intestinal immune network pathway, B cell receptor pathway, T cell receptor pathway, primary immunodeficiency pathway, natural killer cell pathway, cell adhesion molecule pathway and pathways in cancer pathway in Figure 3A. Interestingly, we found that EPAS1 copy number variation (CNV) impacted the infiltration levels of B cells, CD8+ T cells, CD4+ T cells, macrophages, neutrophils and dendritic cells in colon adenocarcinoma (COAD) data (Figure 3B). At the same time, we analysed the correlations between *EPAS1* expression and the levels of immune infiltrating cells in the colorectal tumour. In COAD data, the expression of *EPAS1* was correlated with the expression levels of B cells, CD8+ T cells, CD4+ T cells, macrophages, neutrophils and dendritic cells (Figure 3C). As shown in Figure 3D, *EPAS1* expression was correlated with the levels of CD8+ T cell, macrophages, neutrophils and dendritic cells in rectal adenocarcinoma (READ).

### 3.6 Correlation analysis between *EPAS1* expression and various immune markers

The correlations between *EPAS1* expression and immune signatures were validated using the TIMER database. Immune cells were identified according to the gene markers in Table 3. The values were adjusted based on tumour purity, which affected the dissection of immune infiltration. *EPAS1* expression was associated with the vast majority of immune markers for different types of immune cells in colorectal cancer. The correlations between *EPAS1* expression and multiple markers of T cells were examined. The mRNA levels of *EPAS1* were significantly correlated with 27 of 32 T cell markers in the COAD dataset. In the READ dataset, 19 of 32 T cell markers were correlated with mRNA expression levels of *EPAS1*.

**TABLE 3:** Correlation analysis between *EPAS1* and marker gene in immune cell using TIMER.

Description	Gene markers	COAD (*n* = 457)				READ (*n* = 166)			
		Unadjusted by purity		Adjusted by purity		Unadjusted by purity		Adjusted by purity	
		Cor	*P*	Cor	*P*	Cor	*P*	Cor	*P*
B cell	*CD19*	0.312	***	0.275	***	0.141	0.070	0.054	0.530
	*CD22*	0.455	***	0.428	***	0.291	***	0.188	*
	*CD70*	0.064	0.169	−0.029	0.562	−0.084	0.280	−0.158	0.064
	*CD79A*	0.225	*	0.094	0.429	0.216	**	0.089	0.296
T cell (general)	*CD3D*	0.252	***	0.172	***	0.169	*	0.044	0.605
	*CD3E*	0.336	***	0.283	***	0.279	***	0.156	0.067
	*CD2*	0.313	***	0.265	***	0.278	***	0.187	*
CD8+ T cell	*CD8A*	0.233	***	0.166	***	0.202	**	0.096	0.260
	*CD8B*	0.076	0.104	0.041	0.411	0.093	0.233	0.034	0.695
Monocyte	*CD86*	0.320	**	0.143	0.228	0.424	***	0.395	***
	*CD115 (CSF1R)*	0.426	0.000	0.370	***	0.416	***	0.337	***
TAM	*CCL2*	0.360	***	0.288	***	0.382	***	0.347	***
	*CD68*	0.412	***	0.357	***	0.404	***	0.338	***
	*IL10*	0.301	***	0.252	***	0.259	***	0.205	*
M1 Macrophage	*INOS (NOS2)*	0.111	*	0.089	0.074	0.145	0.062	0.139	0.102
	*IRF5*	0.156	***	0.156	**	0.068	0.381	0.074	0.385
	*COX2 (PTGS2)*	0.359	***	0.332	***	0.369	***	0.396	***
M2 Macrophage	*CD163*	0.393	0.000	0.327	***	0.460	***	0.429	***
	*VSIG4*	0.268	***	0.192	***	0.224	**	0.161	0.059
	*MS4A4A*	0.298	***	0.227	***	0.371	***	0.331	***
	*IRF4*	0.419	***	0.387	***	0.348	***	0.268	**
Neutrophils	*CD66b (CEACAM8)*	−0.093	*	−0.094	0.058	−0.127	0.102	−0.096	0.263
	*CD11b (ITGAM)*	0.295	***	0.235	***	0.302	***	0.239	**
	*CCR7*	0.458	0.000	0.422	***	0.307	***	0.224	**
Natural killer cell	*KIR2DL1*	0.010	0.832	−0.041	0.412	0.079	0.309	0.027	0.753
	*KIR2DL3*	0.001	0.977	−0.033	0.512	0.069	0.374	0.000	0.999
	*KIR2DL4*	0.133	**	0.069	0.163	0.139	0.074	0.075	0.382
	*KIR3DL1*	0.092	*	0.038	0.445	0.125	0.109	0.076	0.377
	*KIR3DL2*	0.185	***	0.137	**	0.118	0.130	−0.027	0.752
	*KIR3DL3*	−0.005	0.911	−0.007	0.888	−0.012	0.874	−0.056	0.515
	*KIR2DS4*	0.075	0.107	0.060	0.228	0.033	0.671	−0.008	0.925
Dendritic cell	*HLA‐DPB1*	0.260	***	0.176	***	0.204	**	0.080	0.348
	*HLA‐DQB1*	0.166	***	0.089	0.072	0.146	0.061	0.080	0.348
	*HLA‐DRA*	0.259	***	0.178	***	0.160	*	0.038	0.655
	*HLA‐DPA1*	0.299	***	0.231	***	0.224	**	0.107	0.208
	*BDCA‐1 (CD1C)*	0.431	***	0.390	***	0.239	**	0.130	0.128
	*BDCA‐4 (NRP1)*	0.520	0.000	0.469	***	0.518	0.000	0.528	***
	*CD11c (ITGAX)*	0.363	***	0.293	***	0.307	***	0.253	**
Th1	*T‐bet (TBX21)*	0.313	***	0.265	***	0.277	***	0.199	*
	*STAT4*	0.357	***	0.302	***	0.409	***	0.334	***
	*STAT1*	0.298	***	0.265	***	0.412	***	0.332	***
	*IFN‐γ (IFNG)*	0.087	0.061	0.030	0.542	0.200	**	0.085	0.319
	*TNF‐α (TNF)*	0.222	***	0.184	***	0.311	***	0.311	***
Th2	*GATA3*	0.373	***	0.322	***	0.285	***	0.232	**
	*STAT6*	0.274	***	0.293	***	0.246	**	0.211	*
	*BATF*	0.192	***	0.127	*	0.125	0.109	0.119	0.162
	*CD294 (GPR44)*	0.138	**	0.134	**	0.076	0.331	0.082	0.336
	*IL13*	0.114	*	0.079	0.113	−0.049	0.530	−0.097	0.254
Tfh	*BCL6*	0.500	0.000	0.460	***	0.377	***	0.401	***
	*IL21*	0.097	*	0.067	0.180	0.068	0.382	0.013	0.875
	*CD185 (CXCR5)*	0.422	***	0.394	***	0.231	**	0.107	0.208
	*CD278 (ICOS)*	0.320	***	0.275	***	0.407	***	0.354	***
Th17	*STAT3*	0.535	***	0.528	***	0.580	0.000	0.613	***
	*CD121a (IL1R1)*	0.628	0.000	0.601	***	0.598	0.000	0.620	***
	*CD194 (CCR4)*	0.455	***	0.420	***	0.321	***	0.227	**
	*CD196 (CCR6)*	0.320	***	0.326	***	0.245	**	0.237	**
	*IL17 (IL17A)*	0.046	0.331	0.056	0.264	0.000	0.999	−0.001	0.992
	*IL21*	0.097	*	0.067	0.180	0.068	0.382	0.013	0.875
	*IL22*	0.068	0.148	0.116	*	0.032	0.686	0.097	0.256
	*IL23R*	0.182	***	0.189	***	0.293	***	0.293	***
Treg	*FOXP3*	0.383	0.000	0.338	***	0.334	***	0.234	**
	*CD25 (IL2RA)*	0.384	***	0.334	***	0.468	***	0.440	***
	*CCR8*	0.413	***	0.358	***	0.408	***	0.345	***
	*STAT5B*	0.393	0.000	0.413	***	0.439	***	0.422	***
	*TGFβ (TGFB1)*	0.384	0.000	0.314	***	0.267	***	0.160	0.060
T cell exhaustion	*PD‐1 (PDCD1)*	0.240	***	0.162	**	0.218	**	0.105	0.219
	*CTLA4*	0.322	***	0.258	***	0.280	***	0.214	*
	*LAG3*	0.221	***	0.153	**	0.208	**	0.123	0.149
	*TIM‐3 (HAVCR2)*	0.299	***	0.220	***	0.307	***	0.240	**
	*GZMB*	−0.095	*	−0.104	*	0.042	0.589	−0.016	0.848

Abbreviations: COAD, colon adenocarcinoma; Cor, R value of Spearman's correlation; READ, rectum carcinoma; TAM, tumour‐associated macrophage; Tfh, Follicular helper T cell; Th, T helper cell; Treg, regulatory T cell.

* *p* < 0.05;

** *p* < 0.01;

*** *p* < 0.001.

**FIGURE 4:**
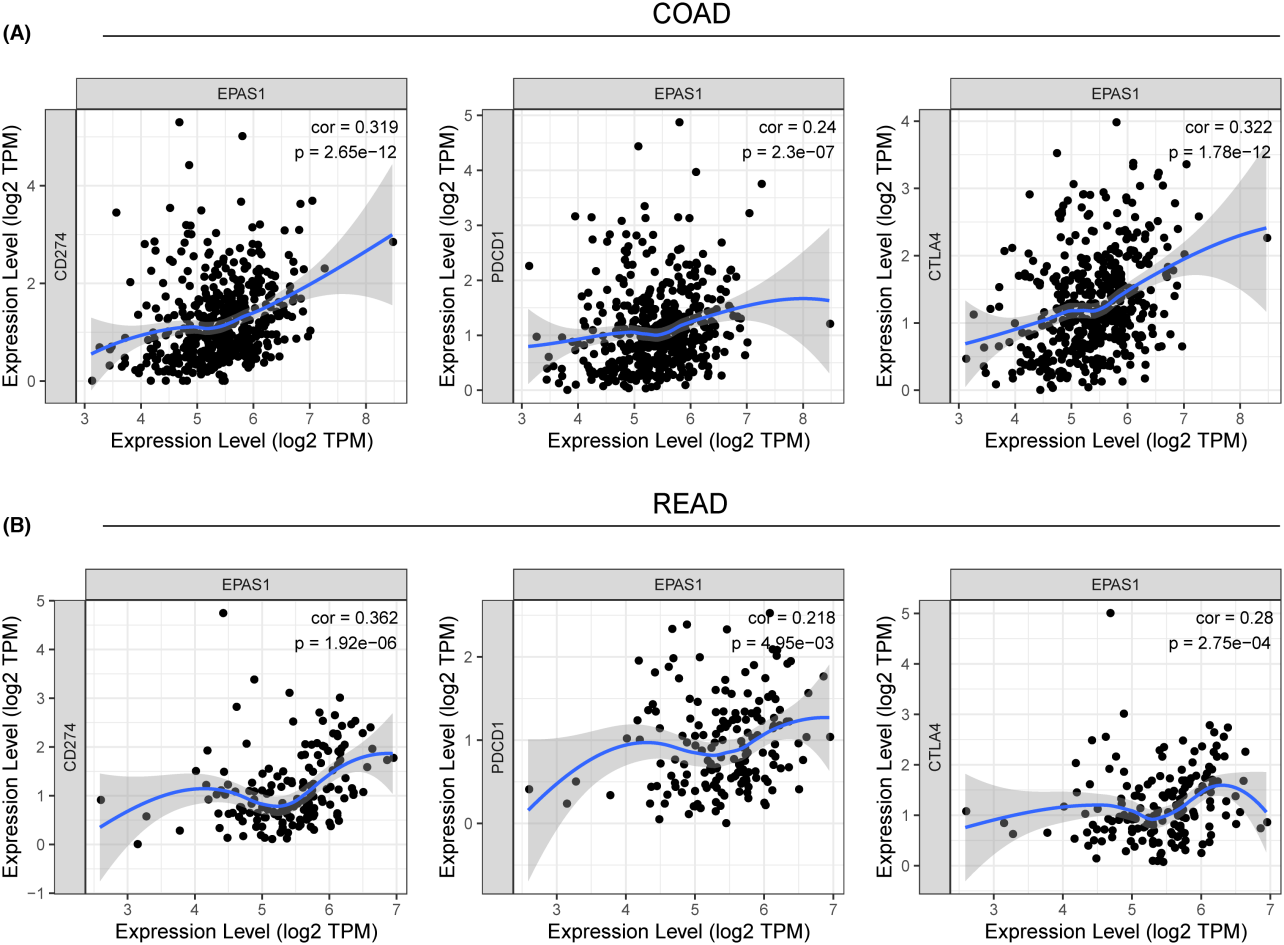
The relationship between *EPAS1* expression and the expression of T cell checkpoints including *PD1*, *PD‐L1* and *CTLA‐4* using the TIMER database. Scatterplots of the correlations between *EPAS1* expression and *PD‐1*, *PD‐L1* and *CTLA‐4* in COAD (A) and READ (B) using the GEPIA database.

Furthermore, the relationship between *EPAS1* expression and the levels of hub T cell checkpoints was investigated. In colorectal cancer, *EPAS1* expression was significantly correlated with the expression of well‐known T cell checkpoints including *PD‐1*, *PD‐L1* and *CTLA‐4* (Figure 4). These findings suggest that *EPAS1* expression is significantly related to immune infiltration and indicated that *EPAS1* may affect immune escape in the microenvironment of colorectal cancer.

## 4 DISCUSSION

In the present study, we investigated the associations between genetic variants in HIF pathway genes and colorectal cancer risk. *EPAS1* rs34533650 significantly increased colorectal cancer risk in the Chinese population, and this result was validated in the Japanese and European populations. *EPAS1* *‐*related genes were enriched in immune‐related pathways, and we found that the mutant of EPAS1 was associated with the degree of infiltration of immune cells. Moreover, significant correlations were observed between *EPAS1* expression and immune marker genes in immune infiltrating cells. These results suggest that *EPAS1* might be a promising predictor of colorectal cancer susceptibility and immune regulation.

The HIF pathway plays a crucial role in tumorigenesis and progression^31^ and causes a series of responses by mediating hypoxia. Hypoxia is linked to diseases such as cancer, diabetes, and inflammation.^32^ Multiple cancers are associated with hypoxia.^33^ The relationships between genetic variants in *HIF‐1α* and diseases have been estimated in a few reported studies. Genetic variants of *HIF‐1α* were connected with colon or rectal cancer risk,^34^ suggesting that genetic polymorphisms in HIF pathway genes may be connected with colorectal cancer. However, few studies analysed the associations between hypoxia‐related genes and colorectal cancer risk, and we identified rs34533650 as a novel colorectal cancer risk factor in the present study. In the Chinese population, compared with the most common haplotype AC, the haplotype GC in the *EPAS1* gene significantly increased colorectal cancer risk. Moreover, SNP rs34533650 increased colorectal cancer risk in two independent replication cohorts, including the Japanese population and European population. However, heterogeneity existed among the three populations. Many GWAS loci are duplicated in different populations but show heterogeneity in effect sizes. The frequencies of the alleles differed, suggesting that the loci are genetically heterogeneous in multiethnic populations, which is probably due to the different population histories and environmental factors of individuals.^35^ The populations of subjects were recruited from different ethnicities, which might result in heterogeneity of different populations. To exclude the effect of heterogeneity, we used the random‐effects model for meta‐analysis.

Colorectal cancer risk can be influenced by multiple factors, and stratified analysis was conducted in different subgroups. Mutant genotypes increased colorectal cancer risk in all subgroups of age. Colorectal cancer has a higher incidence rate in males than in females,^36^ and males with mutant genotypes significantly increased colorectal cancer risk than females. Between rs34533650 and colorectal cancer susceptibility, a more significant association was observed in smokers than non‐smokers. The increased risk was more pronounced in drinkers than in non‐drinkers. Similar phenomena existed in the Dukes stage. However, no significant interaction effects were observed between rs34533650 and demographic characteristics on colorectal cancer risk.

*EPAS1* rs34533650 is located in the intronic region. SNPs in intronic regions may regulate biological activities by dysregulating mRNA splicing, but rs34533650 cannot affect *EPAS1* splicing, indicating that rs34533650 may function in other ways. *EPAS1* rs34533650 can bind the motif domains of BARX1, POU4F2 and POU4F3. POU4F2, as a POU homeodomain transcription factor, promotes the metastatic phenotypes of colorectal cancer.^37^ BARX1^38^ and POU4F3^39^ are both associated with the methylation of cancer. PheWAS are high‐throughput studies, which was used to systematically examine the impact of one or many genetic variants across on a variety of human phenotypes.^40^ After PheWAS analysis, *EPAS1* rs34533650 was associated with multiple diseases, which suggested that rs34533650 is a potential novel biomarker.

EPAS1 is a heterodimeric transcription factor complex.^41^ The predominant regulators of the hypoxic response consist of HIF‐1α and EPAS1, both in cells and organisms.^42^ *EPAS1* is downregulated at the mRNA level in cancerous tissues,^43^ and the same trend was found in colorectal cancer. Similarly, *EPAS1* expression was significantly decreased in colorectal tumour tissues and highly malignant tissues, indicating that *EPAS1* may play an inhibitory role in colorectal cancer risk. To investigate how *EPAS1* affects colorectal cancer, we observed positive correlations between *EPAS1* and angiogenesis, quiescence, differentiation, EMT, metastasis and hypoxia in colorectal cancer at the single‐cell level. These results indicated that *EPAS1* is involved in critical cellular malignant progression, which may impact colorectal cancer.

Hypoxia is related to the immune status of tumour cells. *EPAS1* plays a valuable role in predicting the outcome of PD‐L1 treatment.^9^ SNP rs34533650 influences *EPAS1* expression in an allele‐specific manner, and *EPAS1* expression was associated with the infiltration levels of macrophages,^44^ suggesting that genetic variants may influence immune infiltration through the expression of *EPAS1* gene associated with colorectal cancer risk. Through GSEA, intestinal immune‐associated pathway and cancer‐related pathway were enriched based on *EPAS1* expression in colorectal cancer. Notably, in COAD data, the degree of infiltration of various immune cells was associated with EPAS1 CNV suggesting that genetic variants influence immune status. In single‐cell data, *EPAS1* expression enriched in enteroendocrine cells plays a vital role in intestinal immunity.^45^ In the colorectal tumour, *EPAS1* expression associated with immune infiltrating cells in both COAD and READ data. We also explored the correlations between *EPAS1* expression and immune signatures. Immune cells can be identified by gene markers. *EPAS1* expression was associated with most immune markers in various immune cells. T cells were shown to be associated with immune status,^46^ and the correlations between *EPAS1* expression and multiple T cells were evaluated. *EPAS1* expression level was associated with the vast majority of T cell markers in colorectal cancer data. The *EPAS1* expression was positively correlated with the expression of immune checkpoints for T cells in colorectal cancer. These results suggested that *EPAS1* expression was associated with immune infiltration and indicated that *EPAS1* plays an important role in immune escape in the microenvironment of colorectal cancer.

However, there are some limitations to this study. Lifestyle factors, such as diet and other lifestyle factors, were not collected during the design of this study, so they were not analysed accordingly in the stratified analysis. Molecular biology experiments are required to investigate the specific regulatory mechanisms.

## 5 CONCLUSION

In summary, our research showed that the genetic variant rs34533650 A > G in *EPAS1* was associated with colorectal cancer risk. *EPAS1* affects immune‐related pathways, and the CNV of EPAS1 is related to the infiltration of immune cells. *EPAS1* expression and immune marker genes were significantly correlated in immune infiltrating cells in colorectal cancer. *EPAS1* might be a potential independent biomarker for colorectal cancer risk and immune regulation.

## AUTHOR CONTRIBUTIONS

**Mengfan Guo:** Data curation (equal); writing – original draft (equal). **Jie Lin:** Writing – original draft (equal). **Xiangming Cao:** Data curation (equal); writing – review and editing (equal). **Jieyu Zhou:** Validation (equal); visualization (equal). **Shuai Ben:** Visualization (equal). **Silu Chen:** Software (equal). **Haiyan Chu:** Data curation (equal). **Lin Miao:** Writing – review and editing (equal). **shuwei li:** Writing – review and editing (equal). **Dongying Gu:** Project administration (equal); writing – review and editing (equal).

## FUNDING INFORMATION

This work was supported by the National Natural Science Foundation of China (82073631 and 82103915) and the Natural Science Foundation of Jiangsu Province (BK20210535).

## CONFLICT OF INTEREST STATEMENT

The authors report no conflict of interest.

## Supporting information


Data S1:

## Data Availability

The data that support the findings of this study are available from the corresponding author upon reasonable request.
