# Supplementary material for: Genetic variants in hypoxia‐inducible factor pathway are associated with colorectal cancer risk and immune infiltration
Source: J Cell Mol Med. 2023 Nov 23;28(1):e18019. doi: 10.1111/jcmm.18019 (PMC10805514; doi:10.1111/jcmm.18019)

***Supplementary Material***

**Genetic variants in hypoxia-inducible factor pathway are associated with colorectal cancer risk and immune infiltration**

Mengfan Guo^1, 2, *^, Jie Lin^3, *^, Xiangming Cao^4, *^, Jieyu Zhou^2, 5^, Shuai Ben^2, 5^, Silu Chen^2, 5^, Haiyan Chu^2, 5^, Lin Miao^6, †^, Shuwei Li^2, 5, †^, Dongying Gu^1, †^

^1^ Department of Oncology, Nanjing First Hospital, Nanjing Medical University, Nanjing, China.

^2^ Department of Environmental Genomics, Jiangsu Key Laboratory of Cancer Biomarkers, Prevention and Treatment, Collaborative Innovation Center for Cancer Personalized Medicine, Nanjing Medical University, Nanjing, China;

^3^ The Affiliated Cancer Hospital of Nanjing Medical University, Jiangsu Cancer Hospital, Cancer Institute of Jiangsu Province, Nanjing, China;

^4^ Department of Oncology, The Affiliated Jiangyin Hospital of Nantong University, Wuxi, China;

^5^ Department of Genetic Toxicology, The Key Laboratory of Modern Toxicology of Ministry of Education, Center for Global Health, School of Public Health, Nanjing Medical University, Nanjing, China;

^6^ Medical Center for Digestive Diseases, The second Affiliated Hospital of Nanjing Medical University, Nanjing, China;

^*^These authors contributed equally to this work.

**Supplementary Table 1.** Characteristics of study subjects in this study.

| Variables | Chinese population | |  | Japanese population | |  | European population | |
| --- | --- | --- | --- | --- | --- | --- | --- | --- |
|  | Cases (%), | Controls (%), |  | Cases (%), | Controls (%), |  | Cases (%), | Controls (%), |
|  | n = 1,150 | n = 1,342 |  | n = 6,692 | n = 27,178 |  | n = 4,461 | n = 4,140 |
| Age (mean ± SD) | 59.2 ± 12.8 | 59.2 ± 14.9 |  | 66.9 ± 10.1 | 55.9 ± 10.0 |  | 62.93 ± 10.9 | 60.78 ± 10.0 |
| Sex |  |  |  |  |  |  |  |  |
| Male | 688 (59.8) | 794 (59.2) |  | 4,261 (63.7) | 10,682 (39.3) |  | 2,335 (52.3) | 2,082 (50.3) |
| Female | 462 (40.2) | 548 (40.8) |  | 2,431 (36.3) | 16,496 (60.7) |  | 2,126 (47.7) | 2,058 (49.7) |
| Smoking status |  |  |  |  | |  |  | |
| Never | 751 (65.3) | 901 (67.1) |  | NA | NA |  | NA | NA |
| Ever | 399 (34.7) | 441 (32.9) |  |  | |  |  | |
| Drinking status |  |  |  |  |  |  |  |  |
| Never | 802 (69.7) | 979 (73.0) |  | NA | NA |  | NA | NA |
| Ever | 348 (30.3) | 363 (27.0) |  |  | |  |  | |
| Tumor site |  |  |  |  |  |  |  |  |
| Colon | 586 (51.0) |  |  | NA | |  | NA | |
| Rectum | 564 (49.0) |  |  |  | |  |  | |
| Tumor grade |  |  |  |  |  |  |  |  |
| Well/Moderate | 973 (84.6) |  |  | NA | |  | NA | |
| Poor | 177 (15.4) |  |  |  | |  |  | |
| Dukes stage |  |  |  |  |  |  |  |  |
| I/II | 510 (44.3) |  |  | NA | |  | NA | |
| III/IV | 640 (55.7) |  |  |  | |  |  | |

SD, standard deviation; NA, not available.

**Supplementary Table 2.** Functional annotations between the selected 18 SNPs *in silico* analysis.

| SNPs | Genes | RegulomeDB score | HaploReg v4.1 | | | | | | | Cancer SplicingQTL |
| --- | --- | --- | --- | --- | --- | --- | --- | --- | --- | --- |
|  |  |  | Promoter | Enhancer | DNase | Motifs changed | GRASP QTL hits | Selected eQTL hits | dbSNP func annot |  |
|  |  |  | histone marks | histone marks |  |  |  |  |  |  |
| rs508618 | *EGLN1* | 5 | LNG | 20 tissues | -- | Mrg | -- | 1 hit | intronic | -- |
| rs72704639 | *ARNT* | 5 | -- | GI | -- | 7 altered motifs | -- | 101 hits | intronic | Y |
| rs72992015 | *ARNT* | 5 | -- | FAT, BLD | -- | Pbx-1,Pou2f2,Sox | -- | 35 hits | intronic | -- |
| rs7542797 | *EGLN1* | 5 | 16 tissues | 13 tissues | -- | p300 | 1 hit | 1 hit | intronic | -- |
| rs10847 | *ARNT* | 5 | -- | -- | -- | -- | 3 hits | 53 hits | 3'-UTR | -- |
| rs2121266 | *EPAS1* | 4 | LIV, BRN | ADRL,BLD,BRN,BRST,CRVX,FAT,GI, | ESDR,SKIN,HRT, | -- | -- | -- | intronic | -- |
|  |  |  |  | HRT,LIV,LNG,MUS,OVRY, | GI,LNG,PLCNT,LIV |  |  |  |  |  |
|  |  |  |  | PANC,PLCNT,SKIN,VAS |  |  |  |  |  |  |
| rs34533650 | *EPAS1* | 5 | -- | STRM,FAT,BRST,MUS, | OVRY | 6 altered motifs | -- | -- | intronic | -- |
|  |  |  |  | VAS,GI,OVRY,ADRL,SKIN,PLCNT |  |  |  |  |  |  |
| rs3768727 | *EPAS1* | 4 | -- | FAT,GI,LIV,LNG,BLD,STRM, | ESDR,SKIN | Smad3,Smad4, | -- | 3 hits | intronic | -- |
|  |  |  |  | MUS,SKIN,VAS,BRN,BONE |  | Zfp410 |  |  |  |  |
| rs6720535 | *EPAS1* | 4 | STRM,BRN,FAT,HRT,VAS,GI, | HRT,CRVX,LNG,ESC,IPSC,ESDR,FAT, | ESDR,GI,PLCNT, | -- | -- | -- | intronic | -- |
|  |  |  | OVRY,PANC,LIV,SKIN,LNG,BONE | BRST,BRN,MUS,GI,PLCNT,PANC,VAS,BLD,SKIN | PANC,BRN,SKIN |  |  |  |  |  |
| rs6753127 | *EPAS1* | 3a | -- | PLCNT,LNG,SPLN,BLD | BLD | 4 altered motifs | 1 hit | -- | intronic | -- |
| rs138785226 | *ELOC* | 4 | -- | -- | -- | SIX5 | -- | -- | 3'-UTR | -- |
| rs72661848 | *ELOC* | 5 | -- | -- | -- | 9 altered motifs |  | 1 hit | intronic | Y |
| rs11083817 | *HIF3A* | 3a | 11 tissues | 19 tissues | 14 tissues | HNF4,LBP-1,Zbtb3 | -- | 6 hits | intronic | -- |
| rs11665853 | *HIF3A* | 1f | -- | -- | -- | Irf | 1 hit | -- | intronic | -- |
| rs3764609 | *HIF3A* | 5 | -- | HRT | OVRY,MUS | 6 altered motifs | -- | -- | missense | -- |
| rs3810298 | *HIF3A* | 4 | 15 tissues | 14 tissues | 14 tissues | 8 altered motifs | -- | -- | intronic | -- |
| rs4071668 | *HIF3A* | 4 | -- | SKIN | GI | AP-1,ATF3,Maf | -- | 2 hits | intronic | Y |
| rs73940660 | *HIF3A* | 5 | -- | -- | LNG,MUS | 4 altered motifs |  | 1 hit | intronic | -- |
| rs757638 | *HIF3A* | 4 | -- | ESDR, IPSC, SKIN | -- | 4 altered motifs | -- | 3 hits | intronic | -- |

Chr, chromosome.

--, no significant functions were reported for SNPs.

Y, SplicingQTL were reported for SNPs.

**Supplementary Table 3.** Associations between 18 SNPs in the HIF pathway and colorectal cancer risk in Chinese population.

| Chr | Gene | SNP | Position | Allele^a^ | OR (95% CI) | *P*-value | OR (95% CI)^b^ | *P*-value^b^ | *P*-value^c^ |
| --- | --- | --- | --- | --- | --- | --- | --- | --- | --- |
|  |  |  |  |  |  |  |  |  |  |
| 1 | *EGLN1* | rs508618 | 231532312 | A>G | 0.96 (0.79-1.16) | 6.60×10^-1^ | 0.96 (0.79-1.16) | 6.60×10^-1^ | 7.43×10^-1^ |
| 1 | *EGLN1* | rs72704639 | 150798335 | A>C | 0.92 (0.82-1.04) | 1.98×10^-1^ | 0.92 (0.82-1.04) | 1.98×10^-1^ | 3.96×10^-1^ |
| 1 | *EGLN1* | rs72992015 | 150842696 | C>T | 1.08 (0.90-1.31) | 4.06×10^-1^ | 1.08 (0.90-1.31) | 4.08×10^-1^ | 5.22×10^-1^ |
| 1 | *EGLN1* | rs7542797 | 231553787 | A>C | 1.13 (0.95-1.34) | 1.67×10^-1^ | 1.13 (0.95-1.34) | 1.67×10^-1^ | 3.76×10^-1^ |
| 2 | *EPAS1* | rs2121266 | 46535924 | C>A | 1.07 (0.95-1.20) | 2.88×10^-1^ | 1.07 (0.95-1.20) | 2.94×10^-1^ | 3.99×10^-1^ |
| 2 | *EPAS1* | rs34533650 | 46572449 | A>G | 1.43 (1.20-1.70) | 4.64×10^-5^ | 1.43 (1.20-1.70) | 4.92×10^-5^ | 8.35×10^-4^ |
| 2 | *EPAS1* | rs3768727 | 46590725 | A>G | 1.18 (0.98-1.42) | 8.74×10^-2^ | 1.18 (0.98-1.42) | 8.93×10^-2^ | 2.25×10^-1^ |
| 2 | *EPAS1* | rs6720535 | 46547494 | A>G | 1.07 (0.95-1.21) | 2.75×10^-1^ | 1.07 (0.95-1.21) | 2.83×10^-1^ | 4.13×10^-1^ |
| 2 | *EPAS1* | rs6753127 | 46597296 | C>T | 1.45 (1.17-1.79) | 6.57×10^-4^ | 1.46 (1.18-1.81) | 4.52×10^-4^ | 5.91×10^-3^ |
| 8 | *ELOC* | rs138785226 | 74857541 | A>G | 1.21 (0.98-1.51) | 8.28×10^-2^ | 1.21 (0.97-1.51) | 8.37×10^-2^ | 2.48×10^-1^ |
| 8 | *ELOC* | rs72661848 | 74867927 | C>G | 1.08 (0.95-1.23) | 2.60×10^-1^ | 1.08 (0.94-1.23) | 2.64×10^-1^ | 4.25×10^-1^ |
| 19 | *HIF3A* | rs11083817 | 46803875 | G>A | 1.08 (0.95-1.24) | 2.32×10^-1^ | 1.08 (0.95-1.24) | 2.37×10^-1^ | 4.18×10^-1^ |
| 19 | *HIF3A* | rs11665853 | 46834683 | A>G | 0.98 (0.87-1.10) | 6.85×10^-1^ | 0.98 (0.87-1.10) | 6.81×10^-1^ | 7.25×10^-1^ |
| 19 | *HIF3A* | rs3764609 | 46823702 | A>G | 1.16 (1.03-1.30) | 1.18×10^-2^ | 1.16 (1.03-1.30) | 1.21×10^-2^ | 7.08×10^-2^ |
| 19 | *HIF3A* | rs3810298 | 46800633 | G>A | 1.05 (0.92-1.19) | 4.80×10^-1^ | 1.05 (0.92-1.19) | 4.78×10^-1^ | 5.76×10^-1^ |
| 19 | *HIF3A* | rs4071668 | 46818908 | C>T | 0.82 (0.70-0.96) | 1.63×10^-2^ | 0.82 (0.70-0.96) | 1.60×10^-2^ | 5.87×10^-2^ |
| 19 | *HIF3A* | rs73940660 | 46815993 | T>C | 1.32 (1.06-1.63) | 1.24×10^-2^ | 1.31 (1.06-1.63) | 1.28×10^-2^ | 5.58×10^-2^ |
| 19 | *HIF3A* | rs757638 | 46812126 | G>A | 1.01 (0.87-1.18) | 8.58×10^-1^ | 1.01 (0.87-1.18) | 8.64×10^-1^ | 8.58×10^-1^ |

Chr, chromosome; OR, odds ratio; CI, confidence interval.

^a^Reference allele > effect allele.

^b^*P* for additive model adjusted for age, sex, PC1 and PC2 (where appropriate) in logistic regression model.

^c^*P* after false discovery rate correction.

**Supplementary Table 4.** Associations of combined genotypes of rs34533650 and rs6753127 with the risk of colorectal cancer.

| Risk allele numbers | OR (95% CI)^a^ | *P*^a^ |
| --- | --- | --- |
|  |  |  |
| 0 | 1.00 |  |
| 1 | 1.86 (1.55-2.22) | 1.12×10^-11^ |
| 2-4 | 1.78 (1.25-2.54) | 1.54×10^-3^ |
| *P*-trend | 1.58 (1.38-1.81) | 6.59×10^-11^ |

OR, odds ratio; CI, confidence interval.

^a^ *P* for additive model adjusted for age, sex, PC1 and PC2 (where appropriate) in logistic regression model..

**Supplementary Table 5.** Estimated frequency of haplotypes and association with of colorectal cancer risk.

| Gene | Haplotype^a^ | Estimated frequency | | |  | Effect | |
| --- | --- | --- | --- | --- | --- | --- | --- |
|  |  | Pool | Case | Control |  | OR (95% CI)^b^ | *P*^b^ |
| *EPAS1* | AC | 80.50% | 77.13% | 83.58% |  | 1.00 |  |
|  | AT | 7.19% | 8.52% | 5.97% |  | 1.53 (1.20-1.93) | 4.65×10^-4^ |
|  | GC | 11.54% | 13.52% | 9.78% |  | 1.48 (1.23-1.78) | 3.52×10^-5^ |

OR, odds ratio; CI, confidence interval.

^a^ Haplotypes observed with >1% frequency in pool.

^b^ *P* for additive model adjusted for age, sex, PC1 and PC2 (where appropriate) in logistic regression model.

**Supplementary Table 6.** The MAF in different ethnic populations from the 1000 Genomes Project.

| Population | MAF |
| --- | --- |
| [South Asian](https://www.ncbi.nlm.nih.gov/biosample/SAMN07486027) | G=0.325 |
| [**Europe**](https://www.ncbi.nlm.nih.gov/biosample/SAMN07488239) | **G=0.2237** |
| [**East Asian**](https://www.ncbi.nlm.nih.gov/biosample/SAMN07486024) | **G=0.1081** |
| [American](https://www.ncbi.nlm.nih.gov/biosample/SAMN07488242) | G=0.365 |
| [African](https://www.ncbi.nlm.nih.gov/biosample/SAMN07486022) | G=0.1581 |

MAF, minor allele frequency

**Supplementary Table 7.** Gene-based analysis results by VEGAS2.

| Chr | Gene | Start | Stop | TopSNP | *P-*value | *P*^a^ | *P*^b^ |
| --- | --- | --- | --- | --- | --- | --- | --- |
| 1 | *EGLN1* | 231489496 | 231570790 | rs2808593 | 7.84×10^-3^ | 1.59×10^-3^ | 3.96×10^-3^ |
| 1 | *ARNT* | 150772180 | 150859244 | rs72704639 | 3.73×10^-1^ | 1.98×10^-1^ | 1.98×10^-1^ |
| 2 | *EPAS1* | 46514540 | 46623842 | rs34533650 | 1.94×10^-3^ | 4.64×10^-5^ | 2.32×10^-4^ |
| 8 | *ELOC* | 74847372 | 74894522 | rs4276685 | 2.63×10^-2^ | 7.39×10^-3^ | 1.23×10^-2^ |
| 19 | *HIF3A* | 46790302 | 46856690 | rs3764609 | 9.10×10^-3^ | 1.18×10^-2^ | 1.47×10^-2^ |

Chr, chromosome; nSNPs, number of SNPs.

^a^ after false discovery rate correction.

^b^ *P-*value for topSNP.

**Supplementary Table 8.** Stratification analyses of clinicopathologic variables for the association between rs34533650 and colorectal cancer risk

| Variables |  | OR (95% CI)^a^ | *P*^a^ | *P*_interaction_^b^ | *P*_interaction_^c^ |
| --- | --- | --- | --- | --- | --- |
|  |  |  |  |  |  |
| Age |  | |  |  |  |
| ≤ 60 |  | 1.32 (1.01-1.72) | 4.46×10^-2^ | 2.23×10^-1^ | 8.70×10^-1^ |
| > 60 |  | 1.77 (1.35-2.32) | 3.67×10^-5^ |  |  |
| Sex |  |  |  |  |  |
| Male |  | 1.63 (1.27-2.09) | 1.46×10^-4^ | 8.65×10^-1^ | 9.50×10^-1^ |
| Female |  | 1.40 (1.04-1.87) | 2.69×10^-2^ |  |  |
| Smoking status |  |  |  |  |  |
| Never |  | 1.38 (1.09-1.74) | 6.63×10^-3^ | 2.72×10^-1^ | 9.45×10^-1^ |
| Ever |  | 1.88 (1.34-2.65) | 2.82×10^-4^ |  |  |
| Drinking status |  |  |  |  |  |
| Never |  | 1.42 (1.13-1.77) | 2.21×10^-3^ | 1.78×10^-1^ | 9.29×10^-1^ |
| Ever |  | 1.93 (1.33-2.81) | 5.50×10^-4^ |  |  |

OR, odds ratio; CI, confidence interval.

^a^ *P* for additive model adjusted for age, sex, PC1 and PC2 (where appropriate) in logistic regression model.

^b^ Interaction analysis in the multiplicative model.

^c^ Interaction analysis in the additive model.

**Supplementary Table 9.** Stratification analyses for rs34533650 genotypes and colorectal cancer risk

| Clinical feature | OR (95% CI)^a^ | *P*-value^a^ | *P*-value^b^ |
| --- | --- | --- | --- |
|  |  |  |  |
| Tumour site |  |  |  |
| Colon | 1.57 (1.25-1.98) | 1.01×10^-4^ |  |
| Rectum | 1.48 (1.17-1.87) | 9.96×10^-4^ | 6.37×10^-1^ |
| Tumour grade |  |  |  |
| Well/moderate | 1.56 (1.28-1.90) | 1.17×10^-5^ |  |
| Poor | 1.36 (0.94-1.97) | 1.05×10^-1^ | 5.12×10^-1^ |
| Dukes stage |  |  |  |
| I/II | 1.75 (1.38-2.22) | 3.63×10^-6^ |  |
| III/IV | 1.37 (1.09-1.72) | 6.42×10^-3^ | 8.26×10^-2^ |

OR, odds ratio; CI, confidence interval.

^a^ *P* for additive model adjusted for age, sex, PC1 and PC2 (where appropriate) in logistic regression model.

^b^ Two-sided χ^2^ test for the distributions of genotype and allele frequencies.

**Supplementary Table 10**. The effect of rs34533650 *in silico* prediction.

| SNP | RegulomeDB | |  | 3DSNP v2.0 | |  | HaploReg v4.1 | | |  |
| --- | --- | --- | --- | --- | --- | --- | --- | --- | --- | --- |
|  | Motifs | Score |  | Enhancer | Promotor |  | Enhancer | Motifs | DNase |  |
| rs34533650 | 4 | 5 |  | 14 cell types | NA | | 10 tissues | 6 | 1 tissue |  |

NA, not available.

**Supplementary Table 11**. The PheWAS analysis for rs34533650.

| ID | Trait | Sample size | *P* |
| --- | --- | --- | --- |
| eqtl-a-ENSG00000116016 | ENSG00000116016 | 31,470 | 3.31E-36 |
| eqtl-a-ENSG00000119878 | ENSG00000119878 | 31,684 | 9.47E-31 |
| eqtl-a-ENSG00000250565 | ENSG00000250565 | 31,684 | 1.19E-13 |
| ukb-e-104590_AFR | Other fruit intake | 1,207 | 2.42E-06 |
| eqtl-a-ENSG00000119729 | ENSG00000119729 | 23,842 | 1.67E-05 |
| ukb-b-2102 | Longest period of depression | 64,701 | 1.60E-04 |
| ukb-b-4982 | Able to confide | 448,858 | 1.80E-04 |
| ebi-a-GCST90010321 | Kynurenine-oxoglutarate transaminase 1 levels | 1,313 | 2.24E-04 |
| ebi-a-GCST90002304 | Hematocrit | 562,259 | 2.26E-04 |
| ukb-d-K42 | Diagnoses - main ICD10: K42 Umbilical hernia | 361,194 | 2.29E-04 |
| finn-b-D3_OTHNONTHROMBOCYTOPENPURPURA | Other nonthrombocytopenic purpura | 216,185 | 3.88E-04 |
| ebi-a-GCST90011506 | TestASV_29 (Barnesiella) abundance | 8,956 | 3.99E-04 |
| ukb-b-14541 | Diagnoses - main ICD10: K42.9 Umbilical hernia without obstruction or gangrene | 463,010 | 4.10E-04 |
| ubm-a-596 | IDP dMRI TBSS ISOVF Anterior limb of internal capsule R | 361,194 | 4.17E-04 |
| ukb-d-K61 | Diagnoses - main ICD10: K61 Abscess of anal and rectal regions | 361,194 | 4.94E-04 |
| ebi-a-GCST90010115 | Carcinoembryonic antigenrelated cell adhesion molecule 8 levels | 1,301 | 5.52E-04 |
| prot-a-3067 | Troponin I, cardiac muscle | 3,301 | 5.89E-04 |
| finn-b-I9_LYMPHOTH_EXNONE | Other noninfective disordersof lymphatic vessels and lymph nodes (no controls excluded) | 218,792 | 6.59E-04 |
| ubm-a-2364 | NET100 1208 | 7,916 | 7.08E-04 |
| finn-b-I9_LYMPHOTH | Other noninfective disordersof lymphatic vessels and lymph nodes | 190,427 | 7.35E-04 |
| ukb-e-recode213_CSA | NA | 8,876 | 8.03E-04 |
| ukb-e-recode366_CSA | NA | 8,876 | 8.03E-04 |
| ukb-b-19703 | Low calorie drink intake | 64,949 | 8.20E-04 |
| finn-b-AB1_ASPERGILLOSIS | Aspergillosis | 214,903 | 8.52E-04 |
| ukb-d-30030_irnt | Haematocrit percentage | 350,475 | 8.83E-04 |
| ukb-d-1448_2 | Bread type: Brown | 348,424 | 8.98E-04 |
| ukb-a-470 | Pain type(s) experienced in last month: None of the above | 336,650 | 9.17E-04 |
| ebi-a-GCST90010336 | Dihydropteridine reductase levels | 1,313 | 9.23E-04 |
| finn-b-C3_VULVA_EXALLC | Malignant neoplasm of vulva (all cancers excluded) | 99,446 | 9.56E-04 |
| finn-b-C3_VULVA | Malignant neoplasm of vulva | 123,577 | 9.87E-04 |
| ukb-b-10787 | Standing height | 461,950 | 9.90E-04 |

PheWAS, phenome-wide association study; NA, not available.

**Supplementary Figure legends**

**Supplementary Figure 1. eQTL between candidate SNPs and *EPAS1* from the in-house database.** (A) rs34533650. (B) rs6753127. eQTL, expression quantitative trait loci; SNPs, single nucleotide polymorphisms.

**Supplementary Figure 2. Potential regulatory function of rs34533650.** (**A**) The circular plot of rs34533650 in HCT116 cells. In the circular plot, from the outer circle to the inner circle, the circles represent chromatin states, annotated genes, current SNPs and associated SNPs, and 3D chromatin interactions, respectively. (B) SNP rs34533650 is predicted to affect the combination of BARX1, POU4F2 and POU4F3 using RegulomeDB. SNPs, single nucleotide polymorphisms.

**Supplementary Figure 3. *EPAS1* in 14 crucial functional states at the single-cell level in colon cancer.** Note: **P* ≤ 0.05, ***P* ≤ 0.01.

**Supplementary Figure 4.** *EPAS1* mRNA expression in tumor tissues and normal tissues. (A) Relative expression of *EPAS1* between adjacent tissues, normal tissues, and tumor tissues. (B) Relative expression of *EPAS1* in patients aged ≤ 60 and aged > 60. (C) Relative expression of *EPAS1* at different grades. (D) Relative expression of *EPAS1* at different degrees and ranges of lymph node involvement. (E) Relative expression of *EPAS1* at different stages. (F) Relative expression of *EPAS1* between normal tissues and tumor tissues at different stages.

**Supplementary Figure 5.** **The association of *EPAS1* expression with overall survival based on the GSE12945 database.** Kaplan–Meier survival curve of overall survival based on *EPAS1* expression

**Supplementary Figure 1.**

**
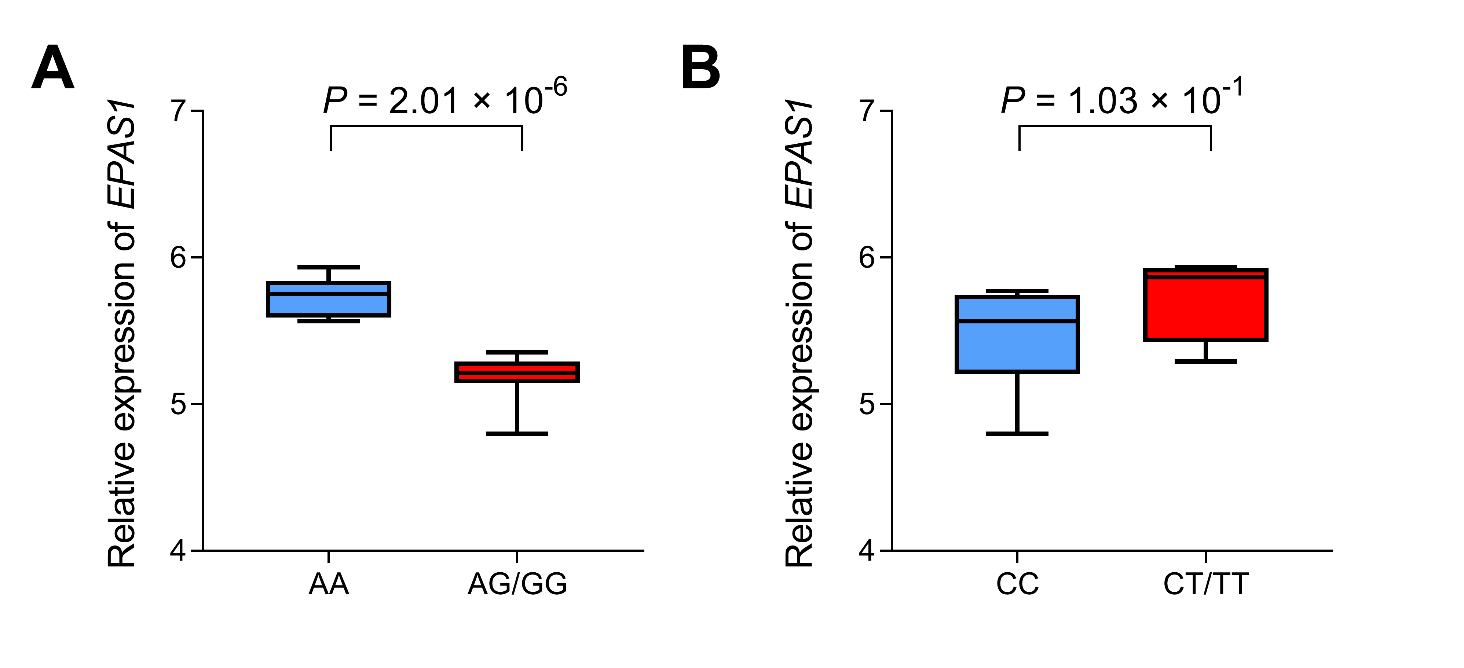
**

**Supplementary Figure 2.**

**
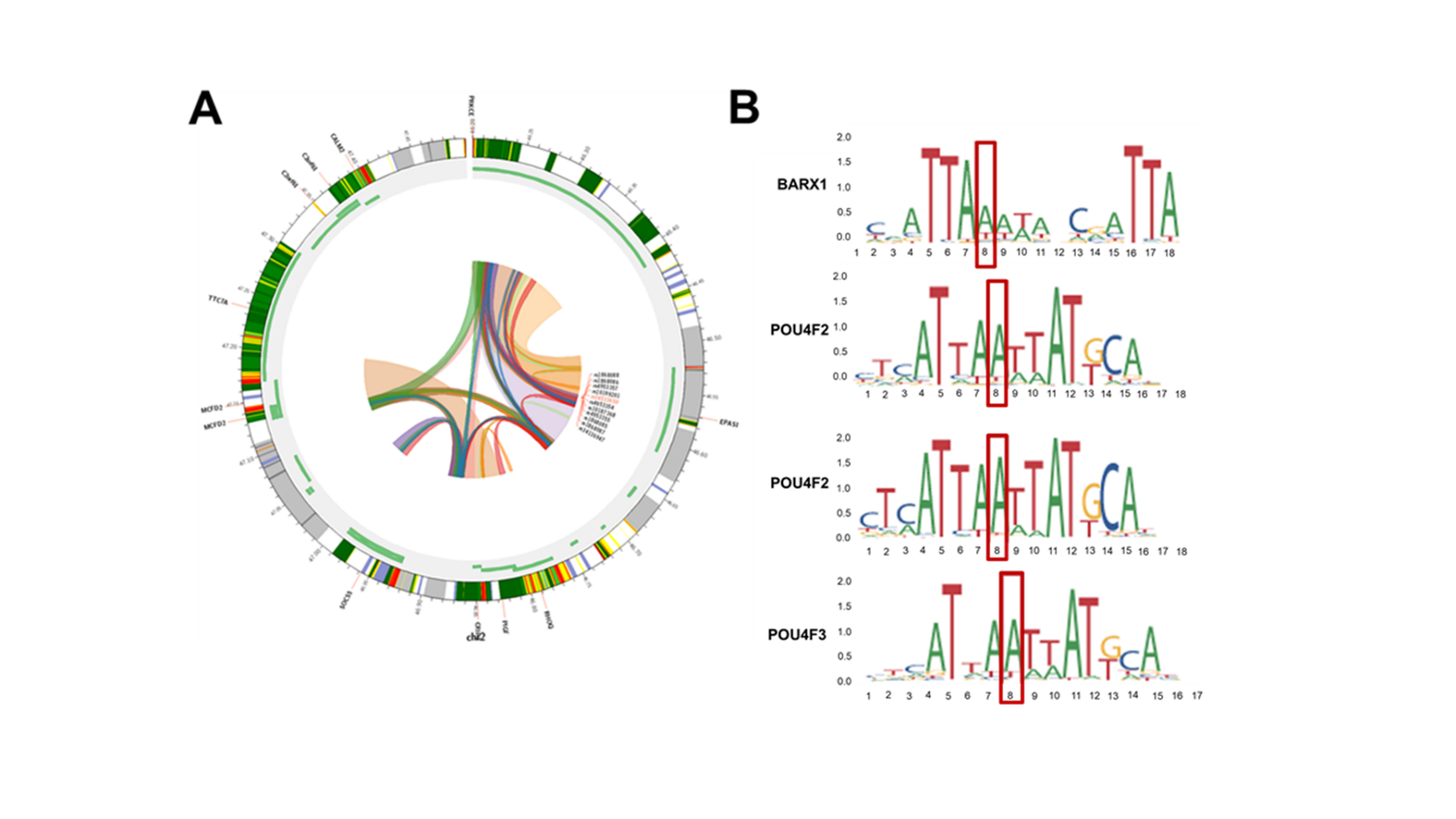
**

**Supplementary Figure 3.**

**
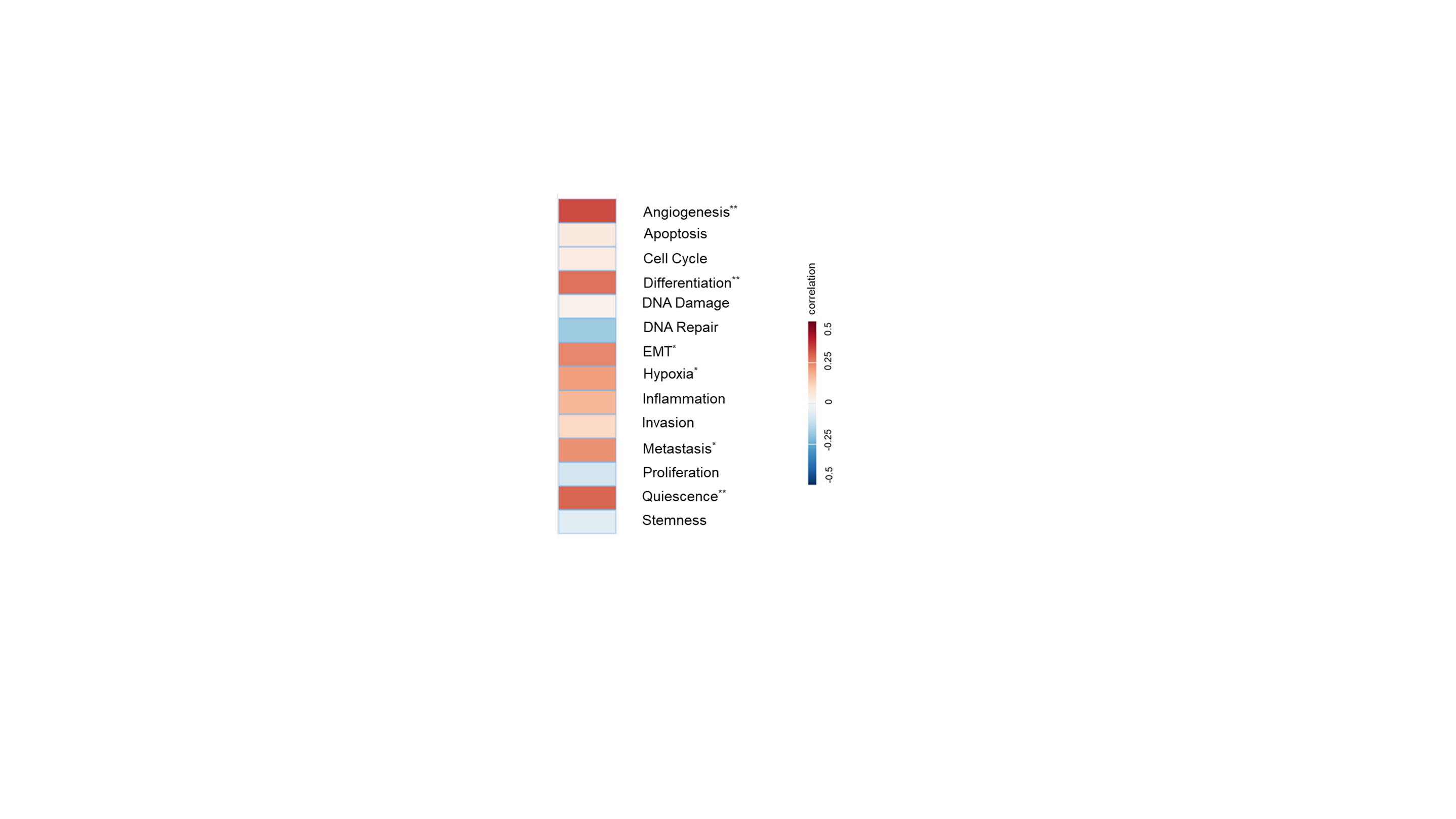
**

**Supplementary Figure 4.**

**
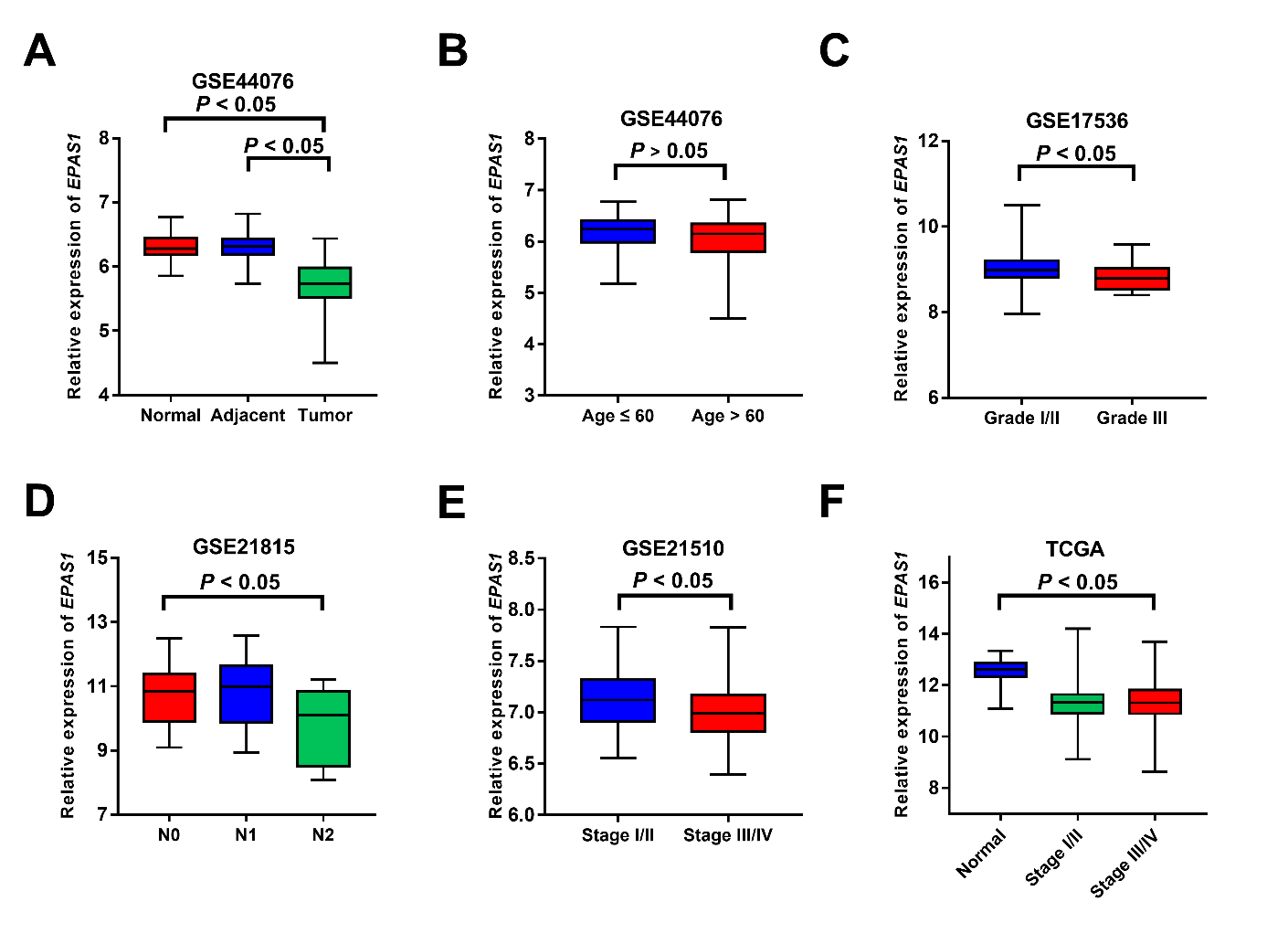
**

**Supplementary Figure 5.**


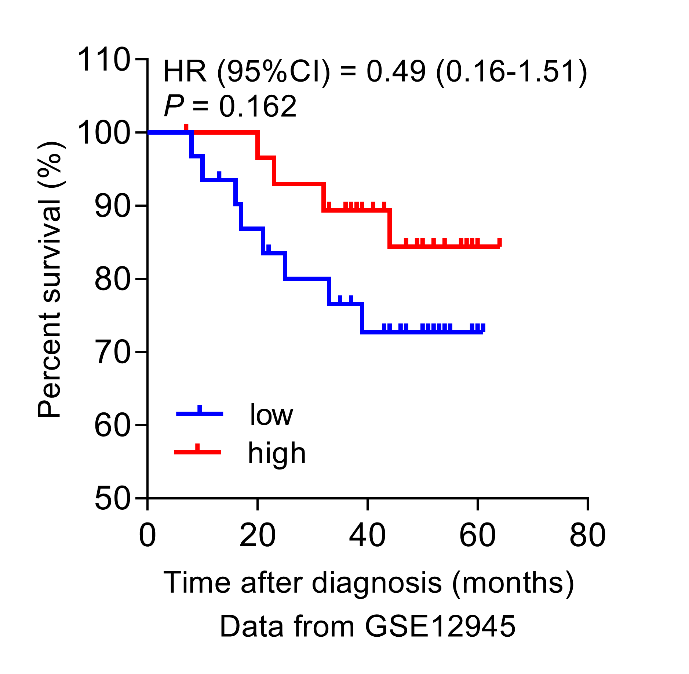

Supplement: Supplementary file 1 — Data S1: [file JCMM-28-e18019-s001.docx]
